# Effect of *Alkanna tinctoria* Root Against MRSA and MDR-*Pseudomonas aeruginosa* Biofilms on Excision Wound in Diabetic Mice: Comparative Study Between Methanolic Extract and Traditional Hydrophobic Preparation

**DOI:** 10.3390/biology13120991

**Published:** 2024-11-29

**Authors:** Yasir Almuhanna, Mohammed Asad, Mohammed S. Alshammari, Babu Joseph

**Affiliations:** Department of Clinical Laboratory Sciences, College of Applied Medical Sciences, Shaqra University, Shaqra 11961, Saudi Arabia; yalmuhanna@su.edu.sa (Y.A.); m.alshammari@su.edu.sa (M.S.A.); bjoseph@su.edu.sa (B.J.)

**Keywords:** cytotoxicity, epithelization, HaCaT cell lines, LCMS analysis, skin irritation

## Abstract

*Alkanna tinctoria*, commonly called dyer’s alkanet (family-*Boraginaceae*), is used traditionally in Saudi Arabia to treat skin infections. A methanolic extract and a traditional formulation of the root used in folklore were prepared. LC-MS analysis was conducted to identify probable compounds present in the extract and the traditional hydrophobic formulation. The in vivo activity on excision wound was evaluated in diabetic mice while crystal violet assay was employed for in vitro evaluation. Human keratinocyte (HaCaT) cells were used to study in vitro cytotoxic effects. Several probable phytoconstituents were revealed by LC-MS analysis in the methanolic extract and the traditional formulation, and three of the constituents were the same. The extract ointment and traditional hydrophobic extract exhibited antibacterial and antibiofilm activity against both tested pathogens. The methanolic extract was relatively more cytotoxic on HaCaT cells compared to the hydrophobic formulation. The methanolic extract ointment did not significantly affect the wound healing, whereas the traditional formulation accelerated wound healing in diabetic mice. The results revealed that *A. tinctoria* in its traditional formulation is an effective wound healing agent but the methanolic extract of the plant does not affect the healing of wounds.

## Simple Summary

*Alkanna tinctoria*, also known as dyer’s alkanet (*Boraginaceae* family), is a traditional plant that has been used to cure skin problems. A traditional hydrophobic extract and a methanolic extract of the root were prepared and the phytocomponents were determined by LCMS analysis. Antibacterial and antibiofilm activities of MRSA and MDR-*Pseudomonas aeruginosa* were determined. Crystal violet assay was used to assess the in vitro antibiofilm activity and a diabetic mice model was used to assess the in vivo antibiofilm activity on excision wounds. In vitro, cytotoxic effects were investigated using human keratinocyte (HaCaT) cells. LC-MS analysis of the methanolic extract and the conventional formulation identified several phytoconstituents, three of which were identical. Against both pathogens, the extract ointment and conventional hydrophobic extract demonstrated antibacterial and antibiofilm action. The methanolic extract was comparatively more cytotoxic than the hydrophobic formulation when tested on HaCaT cells. In diabetic mice, the traditional formulation increased wound healing, but the methanolic extract ointment had no noticeable effect on wound healing. The findings showed that *A. tinctoria* in its conventional form is an effective wound healing agent compared to the methanolic extract that had no effect.

## 1. Introduction

*Alkanna tinctoria *(L.) *Tausch*, belonging to the *Boraginaceae* family, is a flowering plant cultivated in the Mediterranean region. It is primarily grown for its roots, which produce a red dye known as alkanet dye. This dye is used in textiles, cosmetics, and even as food coloring, though its use in food is limited in various regions due to safety concerns [1].

The *A. tinctoria* plant is recognized for its antimicrobial properties. Various extracts from the leaves of *A. tinctoria* have been shown to inhibit the growth of *E. coli* and *S. aureus* [2]. In traditional herbal medicine, alkanet is regarded for its strong anti-inflammatory and wound healing capabilities, despite insufficient validation of its safety and efficacy [3]. The application of *A. tinctoria* red root extracts for coloring and wound treatment has its roots in the eras of Hippocrates and Theophrastus [4]. It is used traditionally in several parts of the world for skin disorders [5,6]. Records from various regions of Greece and the Eastern Mediterranean indicate the sun protection and skin anti-aging properties of the local flora in ethnobotanical and ethnopharmacological studies [7].

Numerous studies have validated the wound healing, antimicrobial, anticancer, and antioxidant effects of root extracts from *A. tinctoria*, which are linked to various compounds [8,9]. The majority of research conducted thus far has focused directly on the chemical composition of *A. tinctoria* and on elucidating the functions of its active ingredients without studying the effects of traditionally used herbal formulations.

There are a few reports on the use of *Alkanet* in the form of an extract or as a traditional formulation that is mentioned in the ancient texts. Alkanet was tested as a single ingredient and in traditional preparation combined with olive oil and beeswax for treating burn wounds clinically. The results indicated a reduction in skin re-epithelialization time [10]. Additionally, a herbal ointment containing extracts from the flower heads of *Matricaria aurea*, aerial parts of *Calendula tripterocarpa*, leaves of *Rosmarinus officinalis*, roots of *A. tinctoria*, and myrrh (an oleo-gum-resin from *Commiphora myrrha*) demonstrated wound healing effects against burn wounds in rats [11]. These reports suggest that traditional herbal formulations are prepared in various ways across different regions. However, the effects of *A. tinctoria* have only been documented concerning burn wounds, with no studies investigating its effect alone or in traditional preparation on wound infection.

Wound infections frequently occur when injured tissue comes into contact with bacteria. Usually, the infectious organism develops a biofilm over the affected area within 24 h, enabling it to escape detection by the immune system and reduce the effectiveness of antimicrobial treatments. Biofilms consist of organized groups of bacterial cells surrounded by a self-generated polymeric matrix made up of polysaccharides, proteins, and nucleic acids [12]. They are considered the main contributors to delayed wound healing because they trigger excessive inflammatory responses that can harm the injured tissue [13]. Consequently, substances designed for wound treatment need to have antimicrobial characteristics as well as the capability to successfully inhibit and remove biofilm formation on damaged tissues [14]. Topical antimicrobial agents are known to cause drug resistance and allergic reactions [15]. Hence, herbal drugs have been used as alternatives to promote the healing of wounds. Furthermore, nanoparticle-based herbal drugs are gaining importance for the treatment of different wounds [16]. The two primary bacteria causing skin infections are Methicillin-resistant *Staphylococcus aureus* (MRSA) and multi-drug-resistant *Pseudomonas aeruginosa *(MDR-*P. aeruginosa*), both of which are recognized for hindering the healing process in diabetic patients [17].

This study investigated the wound healing and antibiofilm effects of *A. tinctoria* root against MRSA and MDR-*P. aeruginosa* infections in diabetic mice. The assessment of antibiofilm efficacy is based on the ethnobotanical belief in Saudi Arabia, which regards this plant as a potent therapeutic agent for wound healing and antimicrobial applications. Therefore, the study examined the antibiofilm effects of the methanolic extract of *A. tinctoria* roots and its traditional formulation used in the central region of Saudi Arabia, which was an olive oil extract of *A. tinctoria* roots and myrrh, an oleo-gum-resin from *Commiphora myrrha* (family-Burseraceae). A detailed phytochemical analysis of both the extract and traditional formulation was also conducted using LC-MS to identify the constituents contributing to the therapeutic wound healing action and any adverse effects on the skin.

## 2. Materials and Methods

### 2.1. Chemicals and Bacterial Culture

All chemicals employed in this study are of analytical grade, unless indicated otherwise. The bacterial pathogens, MRSA (ATCC43300) and MDR-*P. aeruginosa* (ATCC 27853), sourced from the Department of Clinical Laboratory Science at Shaqra University, were utilized in this research.

### 2.2. Extract Preparation

The plant root was collected in August 2023 from the authentic suppliers, and a voucher specimen (No. SU/CAMS/11/2023) is conserved in the department. The dried roots were powdered coarsely, and subjected to Soxhlet extraction using methanol as solvent followed by drying using a rotavapor [18]. The yield of the extract was 13.32% *w*/*w*. Another set of coarsely powdered roots (50 g) was extracted hydrophobically along with myrrh (12 g) using olive oil (375 mL) as the solvent by boiling for 30 min. The preparation was cooled and then filtered using a muslin cloth, and the filtrate was used for further experiments. This was used as a hydrophobic extract.

### 2.3. LC-MS Analysis

The LC-MS analysis for both methanol and hydrophobic extracts was performed using a Waters LC instrument (New Delhi, India) (XEVO-TQD#QCA1232) equipped with a C18 column (SUNFIRE C18, 250 mm × 2.1 mm, 2.6 µm) at a flow rate of 0.2 mL/min and detection set at 280 nm. Acetonitrile served as solvent A, while an ammonium formate buffer was used as solvent B. The HPLC conditions and gradients were consistent with those detailed in our earlier research. Both negative and positive ionization modes were used to record spectra with the *m*/*z* range of 150 to 2000 [19].

### 2.4. Antibacterial Activity (In Vitro)

The broth dilution method was used to determine the minimum inhibitory concentration (MIC) and minimum bactericidal concentration (MBC) [20]. The hydrophobic extract was diluted in a 10% dimethyl sulfoxide solution (Merck KGaA, Germany). The MIC was defined as the lowest concentration that exhibited no visible microbial growth compared to the control, while the MBC was the minimum concentration that led to the complete elimination of viable cells.

### 2.5. Antibiofilm Activity (In Vitro)

The antibiofilm effectiveness of the methanol and hydrophobic extracts was evaluated on both pathogens. Cultures inoculated into Luria–Bertani broth were incubated in a shaking incubator (200 rpm) at 37 °C for 3 h. The optical density (OD at 600 nm) was adjusted to 0.04. From this broth, 100 µL was moved to the wells of a microtiter plate, combined with different sub-MIC concentrations of the extracts, and incubated at 37 °C for 24 h. Following this, the cultures were discarded, and the wells were rinsed thrice carefully with sterile phosphate buffer without affecting the biofilm. A 1% *w*/*v* solution of crystal violet (125 µL) was then added to each well for 1 h to stain the biofilm. The unbound stain was removed followed by washing with the phosphate buffer. Ethanol (70%) was used to dissolve the adhered dye, and the absorbance was measured at 570 nm using an ELISA plate reader [21].

### 2.6. Animals

Swiss albino mice weighing 20 g and approximately 8 weeks old were used in the present study. The experiments were conducted following ARRIVE guidelines [22] and after obtaining ethical approval from the Research Ethics Committee of Shaqra University (Approval No. ERC SU_20220066). A total of 120 animals divided into 10 groups consisting of 12 animals were used.

### 2.7. Extract Formulation and Skin Toxicity

The methanol extract was formulated at two different concentrations of 5% *w*/*w* and 10% *w*/*w* using liquid paraffin, emulsifying wax, and soft paraffin [23]. The hydrophobic oily extract was applied as such on the excised wounds. The physicochemical properties of the prepared ointment were assessed, and the skin irritation test was conducted on the depilated area of the mouse skin [24]. The hydrophobic extract was used for the skin irritation study. The skin area was monitored every 12 h until the 72 h mark for signs of skin toxicity such as irritation or inflammation.

### 2.8. Induction of Diabetes

To induce type II diabetes in mice, streptozocin and nicotinamide were administered using a citrate buffer as the vehicle [25]. Three days after administration of nicotinamide (240 mg/kg, *i.p*) and streptozocin (100 mg/kg, *i.p*), blood glucose levels were measured in 12 h fasted animals. Animals showing a blood glucose level of 150 mg/dL or higher were used for testing the antibiofilm effect. Strict measures were undertaken to prevent the spread of infections.

### 2.9. Antibiofilm Activity In Vivo and Wound Healing

The antibiofilm efficacy of extracts on excision wounds was assessed using a method previously used [26]. A preformed biofilm of MRSA and MDR-*P. aeruginosa* was established on a coverslip. The presence of the biofilm on the coverslip was verified through Gram staining and a crystal violet assay [27]. The animals were given general anesthesia using a cocktail of ketamine and xylazine [28]. The backs of the animals were depilated and an area of 10 mm^2^ was marked. Using a sharp scissor, the skin was carefully excised [26]. A preformed biofilm on the coverslip was placed over the excision wound, along with a small volume of bacterial culture (100 µL with 10^6^ CFU/mL). The wounds were examined for signs of biofilm formation such as the presence of pus and exudate after 72 h. The biofilm layer formed on the wound was confirmed through Gram staining. Following this, the animals were divided into ten groups, with each group containing at least twelve animals until the completion of the experiment. The MRSA-infected animals were grouped as follows:

Group I—applied with the formulation base.

Group II—mupirocin (2%).

Group III and IV—extracts at 5% or 10%, respectively.

Group V received the hydrophobic extract of the oil.

The treatments for MDR-*P. aeruginosa*-infected animals are given below:

Group VI—formulation base.

Group VII—gentamicin ointment (0.1%).

Group VIII and IX were given an application of methanol extract at 5% or 10%, respectively.

Group X received the hydrophobic extract.

The percentage of wound contraction was evaluated by measuring the area every four days. A clear sheet was used to outline the wound, which was then placed over graph paper for area calculation. Mice were euthanized on the 20th day, and the bacterial load in the healed skin (CFU/g) was assessed. Additionally, the newly formed skin was examined histologically with H&E staining. Microscopic changes in the skin tissue were studied under a microscope (Leica DM 2500 LED with DFC 295 camera (Leica Microsystems, Riyadh, Saudi Arabia). The day of scar falling was taken as the epithelization period. 

### 2.10. Cytotoxicity of Extracts on HaCaT Cells

The cytotoxic effects of the extracts on the HaCaT cell line (NCCS, Pune, India) were assessed using the Neutral Red Uptake (NRU) assay. Cells (approximately 7000 per well) were seeded in 96-well plates and cultured for 24 h in Dulbecco’s Modified Eagle Medium (DMEM) (AT149-1L), supplemented with 10% fetal bovine serum (HIMEDIA-RM 10432) and 1% antibiotic solution. The culture was maintained at 37 °C with 5% CO_2_. After 24 h, the culture medium was discarded, and fresh medium was added to each well. The extracts (5 µL) of varying concentrations were then introduced into the designated wells, followed by an additional 24 h incubation. Subsequently, 100 µL of NRU solution (40 µg/mL in PBS) was added to the appropriate wells and incubated in a Heal Force-Smartcell CO_2_ Incubator (Hf-90) for 1 h. Finally, the medium was removed, and NRU was solubilized in 100 µL of NRU destain solution, with absorbance measured at 550/660 nm [29].

### 2.11. Statistical Analysis

The assays and experiments were performed in six trials. The findings were presented as mean values ± standard error of mean and evaluated using one-way analysis of variance (ANOVA), followed by Tukey’s post-test (Statistical Package for the Social Sciences, version 22.0).

## 3. Results

**Figure 1:**
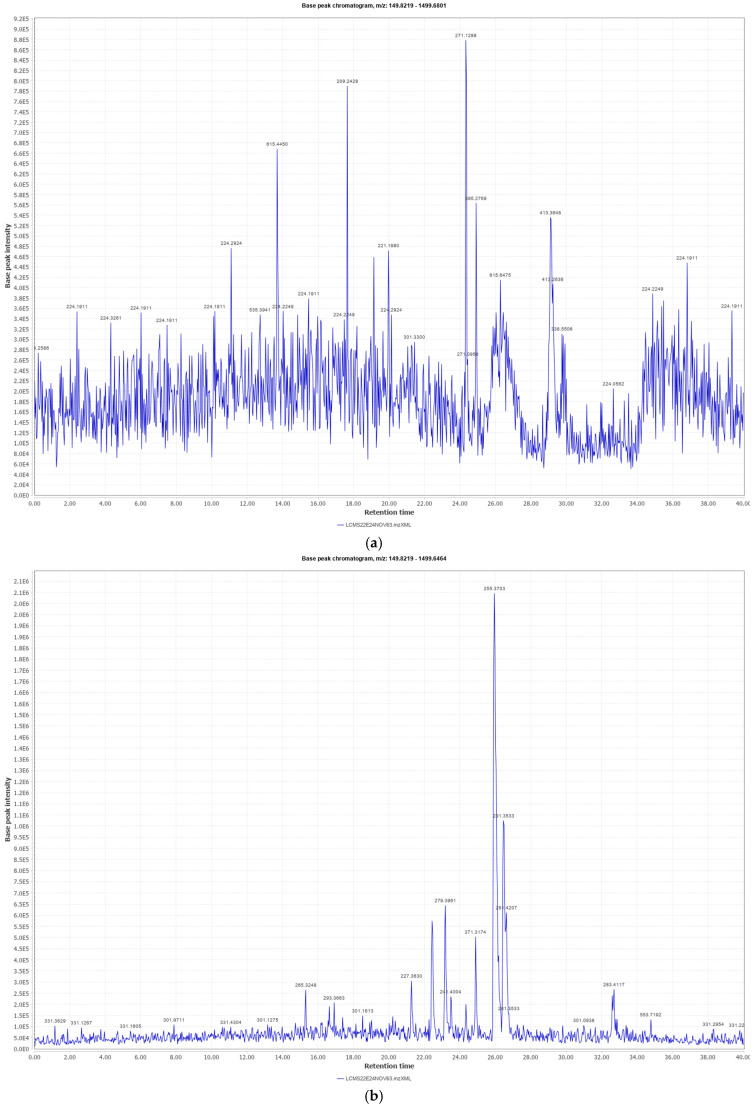
(**a**) Total ion chromatogram in methanol extract (positive mode). (**b**) Total ion chromatogram in methanol extract (negative mode).

**Figure 2:**
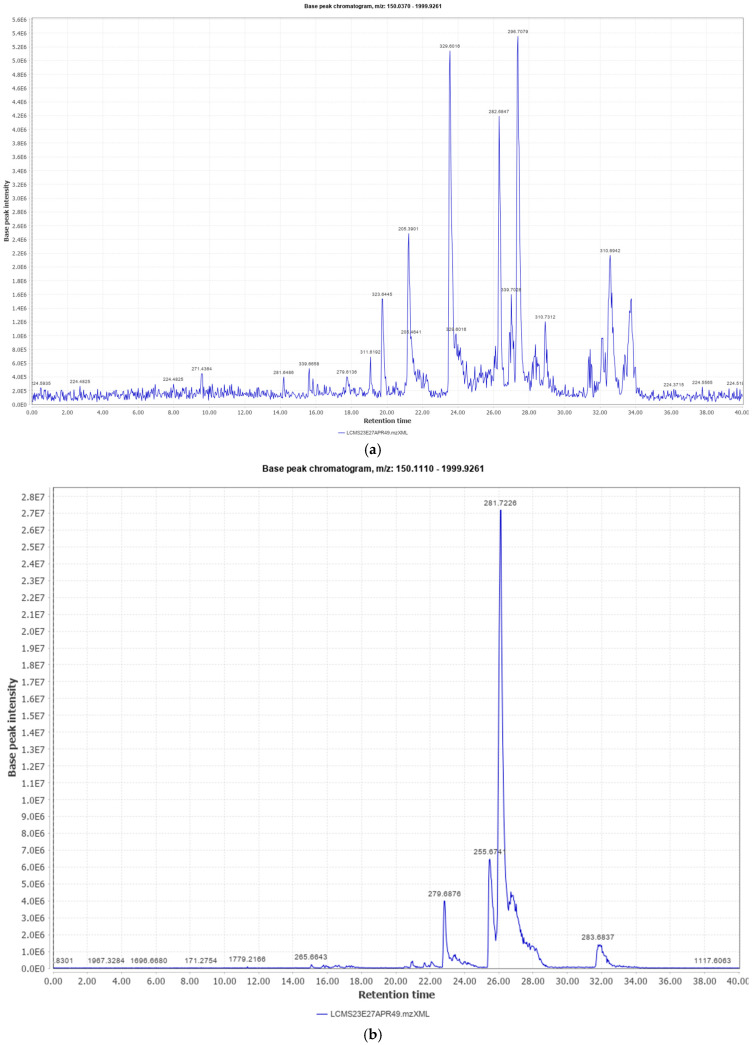
(**a**) Total ion chromatogram in hydrophobic extract (positive mode). (**b**) Total ion chromatogram in hydrophobic extract (negative mode).

The prepared emulsifying ointment was stable and homogeneous, with good diffusion through an agar medium, and a good spreadability at room temperature. Similarly, the traditional hydrophobic extract was found to be stable and homogeneous. LC-MS analysis of both the methanol extract and the hydrophobic extract revealed a wide variety of probable phytoconstituents present in the roots. The total ion chromatograms for the methanol extract in positive mode are displayed in Figure 1a and Table 1a, while those in negative mode are shown in Figure 1b and Table 1b. The total ion chromatogram of the traditional hydrophobic extract for the positive and negative modes are given in Figure 2a and Figure 2b respectively and the list of probable constituents is given in Table 2a,b.

**Figure 3:**
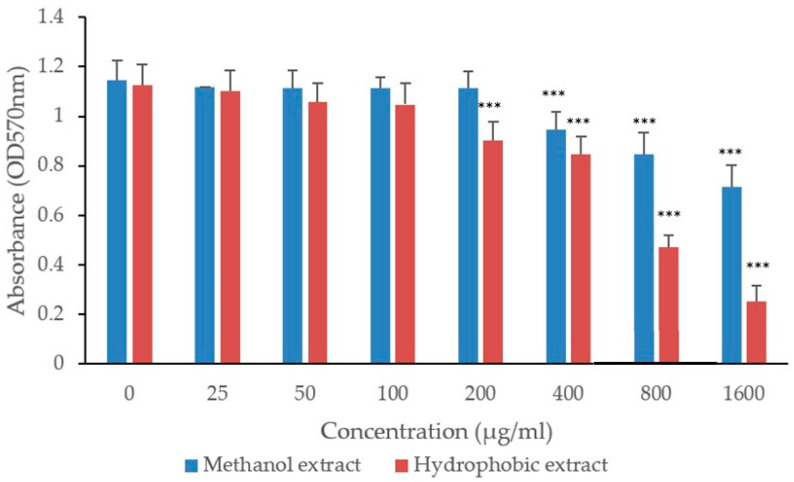
Antibiofilm activity of methanolic and hydrophobic extracts of Alkanna tinctoria against MRSA. Values are mean ± SEM, (n = 6). Asterisks (***) specify statistical difference in *p* < 0.001 compared to control (no treatment).

**Figure 4:**
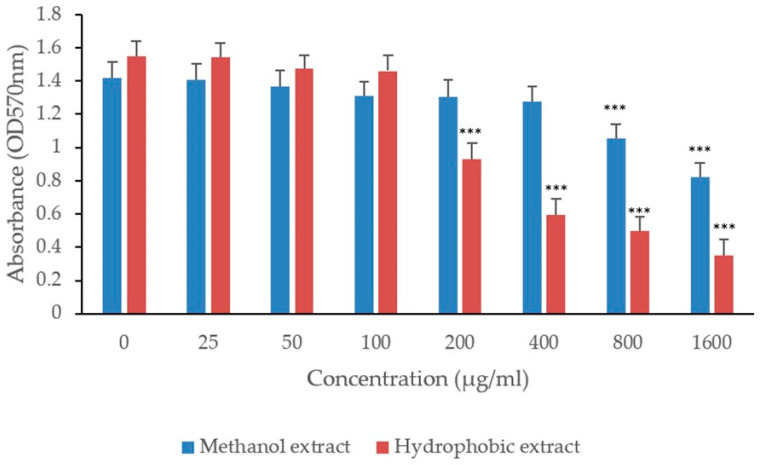
Antibiofilm activity of methanolic and hydrophobic extracts of Alkanna tinctoria against MDR *P. aeruginosa*. Values are mean ± SEM, (n = 6). Asterisks (***) specify statistical difference in *p* < 0.001 compared to control (no treatment).

The extracts demonstrated antibacterial activity against MRSA and MDR-*P. aeruginosa*. The formulated extract was more effective against MRSA than *P. aeruginosa*. Specifically, the methanol extract exhibited a minimum inhibitory concentration (MIC) of 12.5 mg/mL and a minimum bactericidal concentration (MBC) of 25 mg/mL for MRSA. For *P. aeruginosa*, the MIC was 25 mg/mL and the MBC was 50 mg/mL. Similarly, the traditional hydrophobic extract exhibited similar antibacterial values: an MIC of 12.5 mg/mL, an MBC of 25 mg/mL for MRSA, and an MIC of 25 mg/mL and an MBC of 50 mg/mL for *P. aeruginosa*. The antibiofilm efficacy of both methanol and hydrophobic extracts was evaluated at sub-MIC levels against MRSA and MDR-*P. aeruginosa*. Both extracts demonstrated significant antibiofilm activity with effective concentrations starting above 800 µg/mL. Notably, the hydrophobic extract was more potent, inhibiting biofilm formation at just 200 µg/mL. In contrast, the methanol extract inhibited MRSA biofilm formation at 400 µg/mL (Figure 3). For MDR-*P. aeruginosa*, the methanol extract required higher concentrations to achieve similar effects: 400 µg/mL for MRSA and 800 µg/mL for MDR-*P. aerugibosa*. The antibiofilm activity of both extracts appeared to be dose-dependent (Figure 3 and Figure 4).

**Figure 5:**
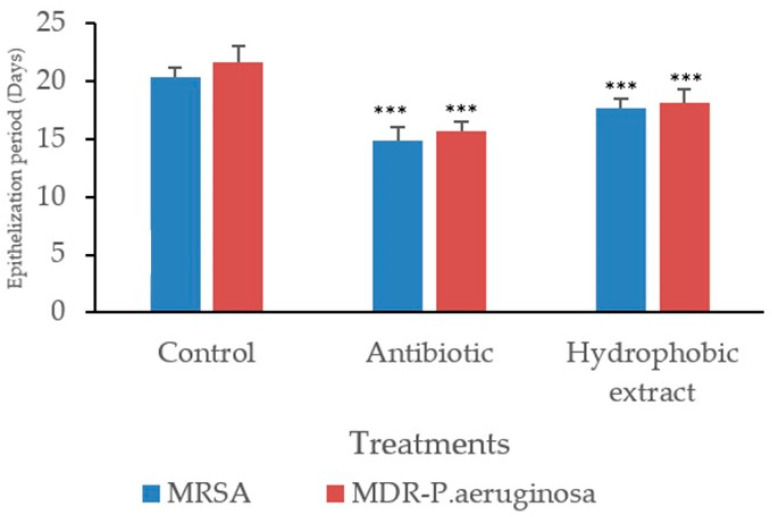
Epithelization period on excision wounds in MRSA and MDR *P. aeruginosa*-induced biofilm-formed wounds. Values are mean ± SEM, (n = 6). Asterisks (***) specify statistical difference in *p* < 0.001 compared to control (base treated).

The ointment formulated with methanol extract was found to be homogeneous and stable and exhibited good spreading properties. Skin irritation tests for both the methanol extract formulation and the traditional hydrophobic extract showed no signs of erythema or inflammation up to 72 h after application. However, in terms of healing efficacy, wounds infected with MRSA displayed a prolonged period of epithelization compared to those infected with MDR *P. aeruginosa* (Figure 5). Even though the methanol extract did not significantly enhance wound contraction or speed up epithelization within 25 days, the hydrophobic extract notably reduced the epithelization period for wounds infected with both MRSA and MDR *P. aeruginosa*.

**Figure 6:**
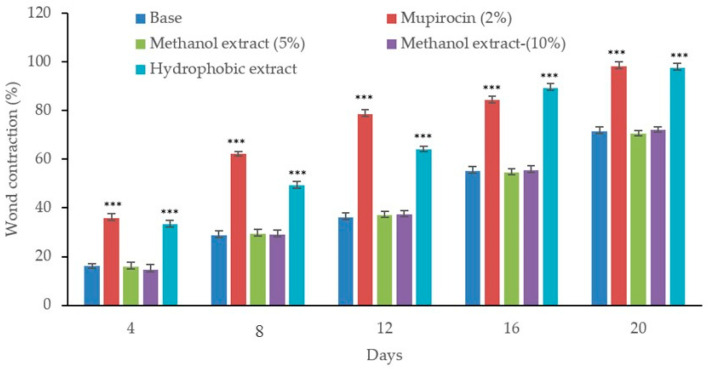
Wound contraction on the excision wound in the MRSA-infected wounds. Wound contraction is indicated by a decrease in the wound area compared to the initial wound area. The values are mean ± SEM, (n = 6). The asterisks (***) specify a statistical difference in *p* < 0.001 compared to the control (base treated).

**Figure 7:**
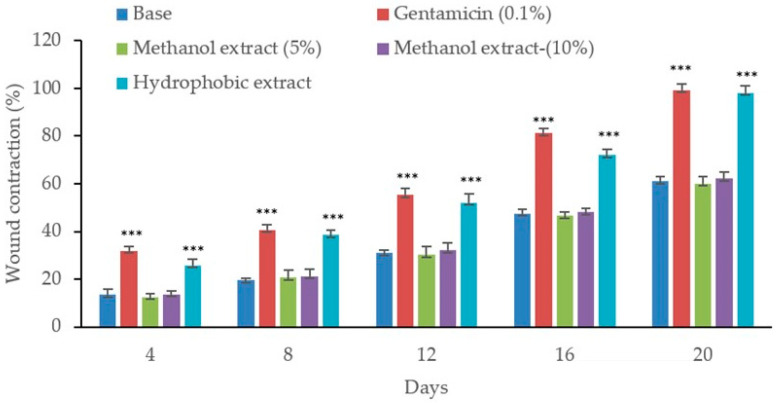
Wound contraction on excision wounds in MDR *P. aeruginosa*-infected wounds. Wound contraction is demonstrated by a decrease in the wound area relative to the initial wound area. The values represent means ± SEM (n = 6). The triple asterisks (***) show statistical difference at *p* < 0.001 compared to the control.

In wound contraction studies, infection with MRSA and MDR *P. aeruginosa* resulted in severe complications, including fluid discharge from the wounds and delayed healing processes. As shown in Figure 6, MRSA infection significantly reduced the healing process. Treatment with the methanol extract did not improve wound contraction, displaying outcomes similar to the control wounds infected with MRSA. The efficacy of the hydrophobic extract in promoting wound healing was observed to be dose-dependent. However, neither the higher concentration (10% *w/w*) nor the lower (5% *w/w*) concentration of the hydrophobic extract significantly affected the wound healing process. Moreover, wound contraction in MRSA-infected wounds treated with the hydrophobic extract was less effective compared to the wound treated with standard antibiotic mupirocin (2%). For wounds infected with, MDR *P. aeruginosa,* the application of the methanol extract at both (5% and 10% *w*/*w*) concentrations appeared not to promote any healing, similar to what was observed in untreated controls. In contrast, it seems that the hydrophobic extract began to show wound healing activity starting from the fourth day in wounds infected with MDR *P. aeruginosa*. Similarly, treatment with gentamicin (0.1%) was observed to start producing significant wound contraction from the fourth day onwards (Figure 7).

After 20 days of treatment, the bacterial load of both MRSA and MDR *P. aeruginosa* in the wounded tissue was assessed. Treatment with the (10%) hydrophobic extract and the methanol extract ointment resulted in a notable reduction in bacterial load compared to the control group, which only received the ointment base. In contrast, the 5% concentration of methanol extract did not significantly decrease the MRSA and MDR *P. aeruginosa* counts. Furthermore, the application of mupirocin and gentamicin on the MRSA-infected wounds and the MDR *P. aeruginosa*-infected wounds, respectively, led to a significant reduction in the bacterial load (Table 3).

**Figure 8:**
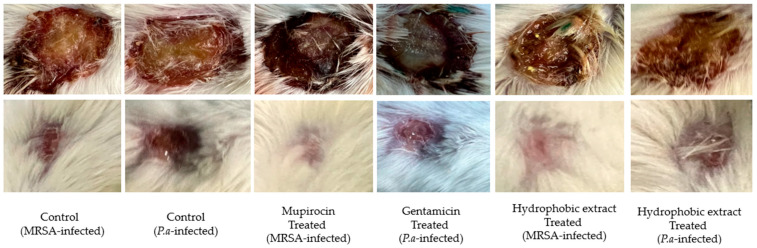
Representative photographs showing excised wounds on Day 0 and Day 20 in different treatment groups. Biofilm formation with pus on Day 0 in all groups. Mupirocin and gentamicin showed best wound healing effects. Photographs from methanolic extract-treated animals not included because no significant effect on wound healing.

**Figure 9:**
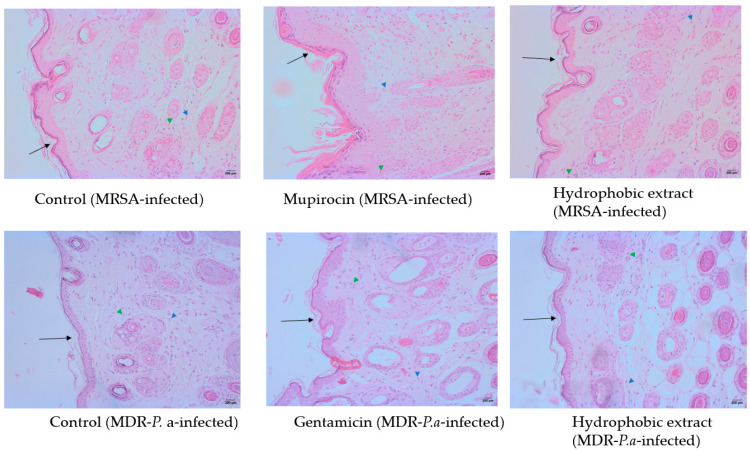
Histological study showing healing of excision wounds (200×) after different treatments. Black arrow indicates regenerated epithelial tissues. Mupirocin showed better healing of MRSA-infected wounds compared to hydrophobic extract. Similarly, gentamicin was more effective than hydrophobic extract on MDR-*P. aeruginosa* infected wounds. Number of inflammatory cells (green arrowhead) lower in treated groups than in control groups. Fibroblasts shown in blue arrowheads. *P.a* indicates MDR-*P. aeruginosa*. Images from methanolic extract-treated group not shown because histology was similar to control group.

Histological analysis post-H&E staining of the injured tissue corroborated the visual observations related to wound healing. The administration of both concentrations of the methanol extract (5 and 10%) appeared to have no effect on the wound. In contrast, the hydrophobic preparation showed a favorable level of skin epithelial tissue regeneration when compared to the control group (Figure 8). These observations of changes in the skin epithelial tissues were made through qualitative microscopic assessments only. The antibiotic treatment for wounds infected with either bacterial pathogen led to complete healing of the tissue, with the regenerated epithelium closely resembling that of normal skin (Figure 9). The methanol extract demonstrated cytotoxic effects on HaCaT cells, indicating it may not be safe for use. Conversely, the hydrophobic extract appeared to be safe, showing no cytotoxic effects on these cells. Additionally, the components utilized in the hydrophobic preparation were also found to be safe as they did not compromise cell viability (Table 4).

## 4. Discussion

The current study investigated the traditional wound healing and antibiofilm effects of *A. tinctoria* root against MRSA and MDR-*P. aeruginosa*-infected wounds in diabetic mice. Motivated by local beliefs in Saudi Arabia, where the plant is reputed as an effective antimicrobial agent, the investigation included both the methanolic extract of *A. tinctoria* roots and also a traditional formulation prepared in the central region of Saudi Arabia. Phytochemical analysis of the extract and the traditional formulation was carried out using LC-MS to identify constituents contributing to the therapeutic wound healing action and the adverse effect on the skin.

The study’s results revealed that the methanolic extract of *A. tinctoria* roots exacerbates wounds infected with MRSA and MDR-*P. aeruginosa* in diabetic mice. In contrast, a traditional preparation of the roots with myrrh, extracted using olive oil, exhibited a positive effect on wound healing. This suggests that *A. tinctoria* is not effective on its own and may require a combination with myrrh, especially when extracted using olive oil, to enhance wound healing. These findings contradict recent studies, such as one by Alam Shah et al., which reported that a chitosan-based combination of mupirocin and *A. tinctoria* root extract improved burn wound healing without causing skin toxicity [9]. Additionally, research by Das et al. indicated that an ethanolic extract of *A. tinctoria* roots demonstrates good antimicrobial and antioxidant properties and is non-toxic [2]. The results indicate that *A. tinctoria* contains both compounds that promote wound healing and those that might hinder it. The choice of extraction solvent is crucial as it can isolate beneficial therapeutic constituents while excluding harmful chemicals. Methanol was used for the extraction of the roots because it extracts several primary and secondary metabolites [30]. The methanol was completely removed from the extract using a rotavapor, which is an effective instrument for the removal of solvents [31]. Furthermore, the findings imply that *A. tinctoria* is effective only when used in combination with olive oil and myrrh to facilitate wound healing.

To identify the molecules suspected of contributing to either the therapeutic or adverse effects observed on mouse skin, we conducted a liquid chromatography-mass spectrometry (LC-MS) analysis of both the crude methanolic extract and the traditional hydrophobic extract. It was found that only three constituents—acacetin, luteolin, and linolenic acid—were present in both extracts. Methanol is capable of extracting most polar constituents from plant materials, while olive oil serves as a nonpolar solvent. This difference accounts for the variations in the constituents found in these extracts. Additionally, the hydrophobic extract contained chemical constituents from both myrrh and olive oil.

Some notable constituents identified in the positive mode of the methanol extract include sinapic acid, methyl jasmonate, nerolidol, farnesol, dihydrozeatin, kynurenine, and salasodine. All of these constituents are reported to possess antioxidant, anti-inflammatory, antimicrobial, and skin cell proliferation activities, which may contribute to enhanced wound healing [32,33,34,35,36].

Pentachlorophenol, a probable compound found in the negative mode, is known to be a pesticide that can aggravate wounds [37]. In contrast, other phytoconstituents such as epicatechin, gamma-linolenic acid, linoleic acid, luteolin, and acacetin exhibit beneficial biological effects that promote wound healing. These compounds exhibit antimicrobial, antioxidant, and anti-inflammatory properties [38,39,40,41].

Four probable compounds identified in the positive mode in the traditional hydrophobic extract—sinapine, carnitine, cyanidin, and kaempferol—are known to enhance wound healing due to their various biological effects [42,43,44,45]. Conversely, the probable constituents scoulerin and safranine have been reported to possess antiproliferative and cytotoxic properties, respectively, with safranine also recognized as a skin irritant [46,47]. Additionally, acacetin, luteolin, and linolenic acid, present in both methanolic and hydrophobic extracts, support wound healing [38,39,41].

From the LC-MS analysis, it is challenging to determine which constituent(s) is responsible for the observed therapeutic wound healing effects in the traditional hydrophobic extract, as well as the pro-wound healing effects seen with the methanolic extract. However, the known cytotoxic and skin irritant effects of safranine and scoulerin suggest their potential role in exacerbating biofilms induced by MRSA and MDR-*P. aeruginosa*.

The preparation method for the hydrophobic extract in this study was different from previous methods [10,11,48]. In a clinical study focusing on burn wounds, a combination of *A. tinctoria*, beeswax, and olive oil was made by adding 30 g of beeswax to 1000 mL of olive oil, which was heated to temperatures between 200 and 210 °C. After the beeswax completely melted, 50 g of *A. tinctoria* (the specific part of the plant used was not mentioned) was incorporated and heated for 5 min. The mixture was subsequently filtered and stored in sterilized bottles. Dressings were prepared just before use by soaking a sterile sponge in the mixture [10]. In a report from the northern part of Saudi Arabia, the traditional formulation was made using methanolic extracts of *Millettia aurea*, *Calendula tripterocarpa*, *Rosmarinus officinalis*, *A. tinctoria*, and myrrh (without extract) were mixed with ointment bases. The amount of each constituent in the ointment was not specified [11]. Another study used a solution of *A. tinctoria* (TAUSCH) at 16% concentration prepared in medical-grade olive oil to enhance the healing of olive oil-burned wounds in rabbits [48]. The exact mechanism underlying the antibiofilm effect of the hydrophobic extract is not known. However, the combination of antibacterial, antioxidant, and wound healing effects of different constituents mentioned above might have contributed to the overall healing action. Furthermore, the emollient effect of the olive oil [49] would have aided in faster wound healing [50].

Previous studies on the wound healing properties of *A. tinctoria* and its formulations have predominantly focused on models of burn wounds without infection. However, infections in all types of wounds are common due to the exposure of wounded tissue to bacteria. As noted earlier, infectious organisms typically form a biofilm over the wounded tissue within 24 h to evade attacks [51]. Biofilms are recognized as a primary cause of delayed wound healing because they trigger inappropriate inflammatory responses that can further damage the tissue [52]. Therefore, evaluating the antibiofilm activities of potential wound healing agents is essential [14]. In this study, we selected two of the most common pathogens associated with skin infections: MRSA and MDR-*P. aeruginosa*. MRSA is implicated in both community-acquired skin and soft tissue infections, as well as nosocomial infections [53,54].

Numerous plant extracts are known to exhibit antibiofilm effects. In Pakistan, *Bergenia ciliata*, * Clematis grata*, and *Clematis viticella* were shown to inhibit *P. aeruginosa* biofilms [55]. African plants like *Alchornea laxiflora* and *Morinda lucida* were also reported to prevent biofilm formation [56], as well as Argentine plants such as *Lycium chilense* [57]. However, most of these reports relied on in vitro methods, which do not provide comprehensive insights into the in vivo effectiveness of these plants [58,59,60]. Furthermore, some active phytoconstituents remain unidentified, complicating the attribution of antibiofilm effects to specific compounds [55,61]. In our study, both the methanolic extract and the traditional hydrophobic extract demonstrated antibacterial and antibiofilm effects in vitro, but these effects were contradictory when tested in vivo. This discrepancy underscores the critical need for in vivo evaluations to accurately assess the biological effects of therapeutic agents.

To validate these effects, our study utilized a mouse model previously identified as effective for studying antibiofilm properties [26]. This approach involved diabetic mice and the application of pre-existing biofilms. The original report evaluated the effect on biofilms caused by MRSA, but it has also been shown to be effective against multidrug-resistant *P. aeruginosa* [62]. The effectiveness in combating biofilm formation was assessed using various measures, including histological evaluations to analyze the healing of excised wounds harboring biofilm. The restoration of skin epithelial tissue after different treatments indicates improved wound healing. The presence of infection and biofilm development interferes with the normal healing process by generating harmful enzymes and toxins, which contribute to chronic inflammation [63]. Factors such as epithelial thickness, capillary density, and collagen content in the wound were employed to assess the wound healing process, with epithelial regeneration being the most frequently evaluated factor [64].

The study has several limitations that can be addressed through future research. While the current investigation identified various chemicals in the extract, a more in-depth analysis involving isolated compounds is necessary to pinpoint the specific chemical constituents responsible for the plant’s antibiofilm and wound healing properties. The findings suggest that other mechanisms, such as antioxidant, anti-inflammatory, and proliferative effects, do not contribute since the wound healing and antibiofilm properties are interconnected. However, the effects of the extract on these other mechanisms have not yet been explored. Although a histological examination was conducted to support both the macroscopic and antimicrobial results, a comprehensive assessment of how the extract influences different processes in wound healing was not performed. Future research could focus on the plant extract’s impact on proliferative genes like vascular endothelial growth factor (VEGF) and transforming growth factor β-1 (TGF-β-1). Additionally, detailed histological studies using various staining methods, such as Masson’s trichrome stain to evaluate collagen deposition, could yield insights into the extract’s proliferative effects [19]. More research is also needed to explore the antioxidant effects by measuring levels of antioxidant enzymes like catalase and superoxide dismutase to assess the plant extract’s free radical scavenging activity [65].

## 5. Conclusions

The methanolic extract of *A. tinctoria* and the traditional hydrophobic extract exhibited different effects on the healing of biofilms induced by MRSA and MDR-*P. aeruginosa* in the murine model. Specifically, the methanolic extract of *A. tinctoria* did not show any effect on the wounded tissue, whereas the hydrophobic extract significantly enhanced wound healing. Furthermore, the antibiofilm activity proved more potent against MRSA than against MDR-*P. aeruginosa.* Both extracts were well tolerated, causing no dermal irritation, and were deemed safe in HaCaT cell line assays. LC-MS analysis of the extracts confirmed the presence of various phytoconstituents, with three constituents namely acacetin, luteolin, and linolenic acid shared between the extracts. These findings underscore the therapeutic potential of employing traditional herbal preparations to maximize clinical benefits.

## Figures and Tables

**Table 1:** (**a**) The phytoconstituents were identified in the methanol extract by total ion chromatogram (positive mode). (**b**) The phytoconstituents in the methanol extract were identified by total ion chromatogram (negative mode).

(a)						
Retention Time	Score	Compound Name	Formula	Exact Mass	Observed Mass	Mass Diff
0.36	0.825	Sinapic acid	C_11_H_12_O_5_	224.068	224.1911	−0.12
1.01	0.761	N-acetyl-D-mannosamine	C_8_H_15_NO_6_	221.089	203.0341	18.05
1.45	0.86	Cystathionine	C_7_H_14_N_2_O_4_S	222.26	224.1574	−1.9
1.52	0.846	cis-Nerolidol	C_15_H_26_O	222.198	224.1911	−1.99
1.62	0.779	cerulenin	C_12_H_17_NO_3_	223.12	224.1574	−1.04
2.03	0.83	Farnesol (mixture of isomers)	C_15_H_26_O	222.198	224.2586	−2.06
2.88	0.797	3-Hydroxy-DL-kynurenine	C_10_H_12_N_2_O_4_	224.21	224.2249	−0.01
5.78	0.853	L-Carnosine	C_9_H_14_N_4_O_3_	226.23	224.1576	2.07
6.02	0.908	3-Hydroxy-DL-kynurenine	C_10_H_12_N_2_O_4_	224.21	224.1911	0.02
10.96	0.58	N-Acetyl-Phytosphingosine	C_20_H_41_NO_4_	359.303	357.5830	1.72
13.69	0.762	Glutathione (oxidized form)	C_20_H_32_N_6_O_12_S_2_	612.63	615.4450	−2.82
18.98	0.717	Cystathionine	C_7_H_14_N_2_O_4_S	222.26	224.1911	−1.93
19.97	0.728	DL-Dihydrozeatin	C_10_H_15_N_5_O	221.127	221.1889	−0.06
22.33	0.738	pelargonidin chloride	C_15_H_11_O_5_	271.06	271.1288	−0.07
24.91	0.71	delta-Tocotrienol	C_27_H_40_O_2_	396.302	395.2769	1.03
26.27	0.603	cyanidin-3,5-di-O-glucoside chloride	C_27_H_31_O_16_	611.161	615.6475	−4.49
29.01	0.618	Solasodine	C_27_H_43_NO_2_	413.329	413.3643	−0.04
29.75	0.697	5-Aminoimidazole-4-carboxamide-1-beta-D-ribofuranosyl 5′-monophosphate	C_9_H_15_N_4_O_8_P	338.062	338.5506	−0.49
34.46	0.864	Methyl Jasmonate	C_13_H_20_O_3_	224.141	224.1574	−0.02
**(b)**						
**R. Time**	**Score**	**Compound Name**	**Formula**	**Exact Mass**	**Observed Mass**	**Mass Diff**
15.31	0.79	Pentachlorophenol	C_6_HCl_5_O	266	265.3248	0.68
16.91	0.811	(+)-Epicatechin	C_15_H_14_O_6_	290	293.3663	−3.37
21.28	0.895	2′-Deoxycytidine	C_9_H_13_N_3_O_4_	227	227.3630	−0.36
22.44	0.888	D-Arabinose-5-phosphate disodium salt	C_5_H_11_O_8_P	230	253.3794	−23.38
23.19	0.969	gamma-Linolenic acid	C_18_H_30_O_2_	278	279.3961	−1.4
24.89	0.784	Lignoceric Acid	C_24_H_48_O_2_	368	371.3174	−3.32
25.95	0.99	D-Glucosamine-6-phosphate sodium salt	C_6_H_14_NO_8_P	259	255.3703	3.63
26.46	0.957	Luteolin	C_15_H_10_O_6_	286	281.3533	4.65
32.70	0.886	Acacetin	C_16_H_12_O_5_	284	283.4117	0.59

**Table 2:** (**a**) Phytoconstituents in hydrophobic extract identified by total ion chromatogram (positive mode). (**b**) Phytoconstituents in hydrophobic extract identified by total ion chromatogram (negative mode).

(a)						
R. Time	Score	Compound Name	Formula	Exact Mass	Observed Mass	Mass Diff
15.62	0.596	Docosanoic acid	C_22_H_44_O_2_	340.334	339.6658	0.6682
21.23	0.872	L-Tryptophane	C_11_H_12_N_2_O_2_	204.23	205.3901	−1.1601
21.29	0.86	O-Acety-L-carnitine hydrochloride	C_9_H_18_NO_4_	204.123	205.4271	−1.3041
21.50	0.732	Etidronic acid	C_2_H_8_O_7_P_2_	205.974	205.4641	0.5099
23.51	0.671	Adenosine-3′,5′-cyclicmonophosphate	C_10_H_12_N_5_O_6_P	329.052	329.6016	−0.5496
23.55	0.689	Hydroxypyruvic acid dimethyl ketal phosphate tri(cyclohexylammonium) salt	C_5_H_11_O_8_P	230.019	329.6016	−99.5826
23.89	0.708	Scoulerin	C_19_H_21_NO_4_	327.147	329.6016	−2.4546
24.13	0.714	Adenosine-3′,5′-cyclicmonophosphate	C_10_H_12_N_5_O_6_P	329.21	329.6016	−0.3916
26.11	0.626	Palmitoleic acid	C_16_H_30_O_2_	254.224	256.5991	−2.3751
26.31	0.953	Acacetin	C_16_H_12_O_5_	284.068	282.6847	1.3833
26.89	0.84	pelargonidin chloride	C_15_H_11_O_5_	271.06	270.6224	0.4376
27.00	0.711	Sinapoyl malate	C_15_H_16_O_9_	340.079	339.7028	0.3762
27.07	0.714	Formononetin	C_16_H_12_O_4_	268.073	265.6643	2.4087
27.34	0.972	5′-Deoxy-5′-Methylthioadenosine	C_11_H_15_N_5_O_3_S	297.089	296.7449	0.3441
28.36	0.603	1-O-b-D-glucopyranosyl sinapate	C_17_H_22_O_10_	386.121	391.7628	−5.6418
28.91	0.836	Sinapine	C_16_H_24_NO_5_	310.165	310.7312	−0.5662
31.44	0.772	Zeatin-9-glucoside	C_16_H_23_N_5_O_6_	381.164	381.8096	−0.6456
32.09	0.792	Folic acid, approx	C_19_H_19_N_7_O_6_	441.139	441.7878	−0.6488
32.67	0.907	Safranine	C_20_H_19_N_4_	315.16	310.7312	4.4288
33.66	0.879	cyanidin-3-O-rhamnoside chloride	C_21_H_21_O_10_	433.113	435.7937	−2.6807
33.76	0.923	1-Lauroyl-2-Hydroxy-sn-Glycero-3-Phosphocholine	C_20_H_42_NO_7_P	439.269	435.7937	3.4753
33.86	0.863	Kaempferol-7-O-alpha-L-rhamnoside	C_21_H_20_O_10_	432.105	435.6827	−3.5777
**(b)**						
**R. Time**	**Score**	**Compound Name**	**Formula**	**Exact Mass**	**Observed Mass**	**Mass Diff**
22.81	0.974	gamma-Linolenic acid	C_18_H_30_O_2_	278.43	279.7246	−1.2946
22.85	0.965	6-Phosphogluconic acid Barium salt hydrate	C_6_H_13_O_10_P	276.024	279.6876	−3.6636
22.88	0.96	L-saccharopine	C_11_H_20_N_2_O_6_	276.132	279.7246	−3.5926
25.41	0.975	2′-Deoxyinosine	C_10_H_12_N_4_O_4_	252.085	255.6741	−3.5891
25.44	0.971	D-Glucosamine-6-phosphate sodium salt	C_6_H_14_NO_8_P	259.045	255.6741	3.3709
25.48	0.961	alpha-D-glucose-1-phosphate dipotassium salt dihydate	C_6_H_13_O_9_P	260.029	255.6741	4.3549
25.58	0.971	2′-Deoxyinosine	C_10_H_12_N_4_O_4_	252.085	255.6741	−3.5891
25.68	0.972	D-Glucosamine-6-phosphate sodium salt	C_6_H_14_NO_8_P	259.045	255.6741	3.3709
26.09	0.967	Acacetin	C_16_H_12_O_5_	284.068	281.7226	2.3454
26.13	0.972	Luteolin	C_15_H_10_O_6_	286.047	281.7226	4.3244
26.71	0.957	Acacetin	C_16_H_12_O_5_	284.068	281.6856	2.3824
26.84	0.961	Xanthosine	C_10_H_12_N_4_O_6_	284.075	281.7226	2.3524
26.91	0.94	Luteolin	C_15_H_10_O_6_	286.047	281.7226	4.3244
27.01	0.964	Acacetin	C_16_H_12_O_5_	284.068	281.6656	2.4024

**Table 3:** Bacterial load in wounded tissue.

	Log_10_ CFU/g of Tissue	
Treatment	MRSA	* P. aeruginosa *
Base	4.934 ± 0.965	5.287 ± 1.256
Antibiotic	1.591 ± 0.234 ***	1.045 ± 0.34 ***
Methanol extract (5%)	4.641 ± 1.052	4.942 ± 0.952
Methanol extract (10%)	2.782 ± 0.996 **	3.524 ± 0.972 *
Hydrphobic extract	1.954 ± 0.842 ***	2.041 ± 0.972 ***

Values are mean ± SEM, n = 6, * *p* < 0.05, ** *p* < 0.01, *** *p* < 0.001 compared to control.

**Table 4:** IC_50_ values of methanolic and hydrophobic extracts.

Sample	IC_50_ Value
Methanolic extract of *A. tinctoria*	0.2736 µg/mL
Traditional hydrophobic extract	0.595 µg/mL
Olive Oil	20.27% *v*/*v*
Myrrh	21.26 µg/mL

## Data Availability

The raw data supporting the conclusions of this article will be made available by the authors on request.
